# Epigenetic DNA Methylation and Protein Homocysteinylation: Key Players in Hypertensive Renovascular Damage

**DOI:** 10.3390/ijms252111599

**Published:** 2024-10-29

**Authors:** Lu Ren, Sathnur Pushpakumar, Hebah Almarshood, Swapan K. Das, Utpal Sen

**Affiliations:** 1 Department of Physiology, University of Louisville School of Medicine, Louisville, KY 40202, USA; lu.ren@cchmc.org (L.R.);; 2 Department of Internal Medicine, Section on Endocrinology and Metabolism, Wake Forest University Health Sciences, Winston-Salem, NC 27157, USA

**Keywords:** epigenetics, DNA methylation, histone modification, noncoding RNA, protein homocysteinylation, metabolic disorder, gaseous molecules, kidney disease

## Abstract

Hypertension has been a threat to the health of people, the mechanism of which, however, remains poorly understood. It is clinically related to loss of nephron function, glomerular sclerosis, or necrosis, resulting in renal functional declines. The mechanisms underlying hypertension’s development and progression to organ damage, including hypertensive renal damage, remain to be fully elucidated. As a developing approach, epigenetics has been postulated to elucidate the phenomena that otherwise cannot be explained by genetic studies. The main epigenetic hallmarks, such as DNA methylation, histone acetylation, deacetylation, noncoding RNAs, and protein N-homocysteinylation have been linked with hypertension. In addition to contributing to endothelial dysfunction and oxidative stress, biologically active gases, including NO, CO, and H_2_S, are crucial regulators contributing to vascular remodeling since their complex interplay conducts homeostatic functions in the renovascular system. Importantly, epigenetic modifications also directly contribute to the pathogenesis of kidney damage via protein N-homocysteinylation. Hence, epigenetic modulation to intervene in renovascular damage is a potential therapeutic approach to treat renal disease and dysfunction. This review illustrates some of the epigenetic hallmarks and their mediators, which have the ability to diminish the injury triggered by hypertension and renal disease. In the end, we provide potential therapeutic possibilities to treat renovascular diseases in hypertension.

## 1. Introduction

Hypertension is referred to as a condition with a systolic blood pressure of at least 140 mm Hg and/or a diastolic blood pressure of at least 90 mm Hg [1]. It contributes to a massive disease burden influencing over one billion people in the world, and, in a 2017–2018 survey report, it was shown that about 50% of the adult population is affected by hypertension in the United States [2]. Hypertension has been recognized as a risk factor for developing stroke, myocardial infarction, heart failure, and end-stage renal disease. Typically, essential hypertension is considered a condition with an unapparent single cause [3]. Arterial blood pressure results from cardiac output and total peripheral vascular resistance, which is regulated by several physiological and neurohormonal mechanisms. These mechanisms involve the immune systems and several organ systems, such as the kidney, cardiovascular, nervous, and endocrine [4].

Hypertensive nephropathy (HN) is defined as chronic kidney disease (CKD) of unknown cause in hypertensive patients, and this definition of HN is, however, non-specific [5]. The reason is that the majority of CKD patients develop hypertension, and kidney biopsy studies did not find specifics for HN, i.e., biopsy results did not establish that hypertension is causative to renal disease in these patients [5,6]. Nevertheless, it is well recognized that over the course of hypertension, kidney damage occurs at various levels, including glomerular, tubular, and interstitial injuries, leading to renal dysfunction [7,8]. The molecular mechanisms underlying hypertensive renal damage and dysfunction include various factors, including but not limited to sympathetic activation, the renin–angiotensin–aldosterone system (RAAS), oxidative stress, endothelial dysfunction, structural abnormalities of vessels, and genetic and epigenetic determinants [8,9]. In this review, we will focus on mostly epigenetic determinants that contribute to hypertensive renal damage and dysfunction.

Vast evidence collected from genome-wide association studies has identified a relationship between genetic variants (single nucleotide polymorphisms/SNPs) and increased arterial blood pressure. Nonetheless, those genetic variants explain only a tiny portion of phenotypic variation, and the role of those SNPs and their target genes in the pathogenesis of hypertension is obscure [10,11,12]. For a broader exploration and a deeper understanding of the molecular processes of hypertension, epigenetic mechanisms have been brought into the spotlight for their function in gene expression regulation, which is potentially modifiable by environmental factors [13]. This review integrates hypertension-associated epigenetic effects on tissues, particularly on the endothelium, tubule, glomerulus, and kidney, and outlines its significant role in the pathogenesis of kidney diseases. The prime epigenetic means are highlighted. A variety of epigenetic marks and mediators are tested in an attempt to seek the links between kidney damage under hypertension and the promising epigenetic modification strategies to diminish the injury triggered by hypertension.

## 2. Epigenetics of Disease Mechanism

**Figure 1:**
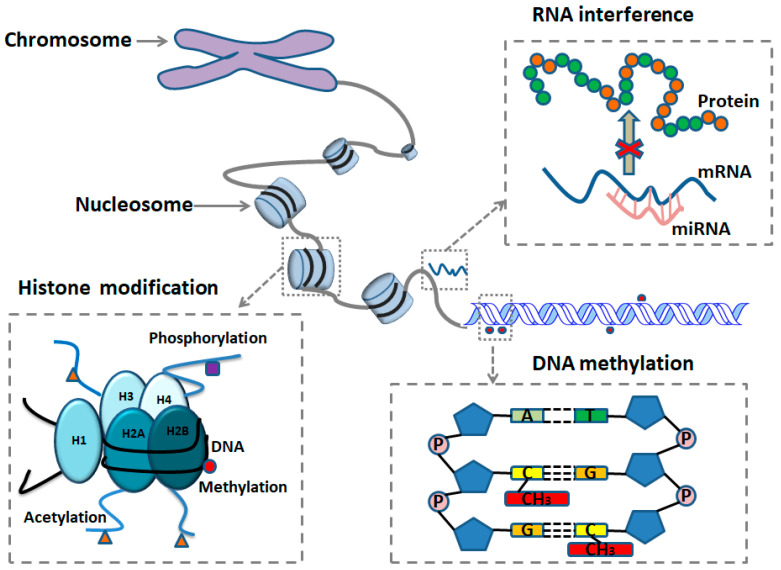
Major mechanisms of epigenetic modification. DNA methylation is a covalent binding of a methyl group to 5′ carbon of cytosine (C) located at a CpG dinucleotide, which generally prevents a gene’s transcription when taking place in the gene promoter. Histone modifications are covalent post-translational alterations of N-terminal tails of four core histones, including H3, H4, H2A, and H2B, affecting gene expression by controlling the conformation and dynamics of chromatin. RNA interference refers to noncoding RNAs, including small noncoding RNAs (miRNAs) that have a downregulating influence on gene transcription by causing target mRNA degradation or by mRNA translational repression.

Epigenetics refers to functional alterations in gene expression that leave the heritable DNA sequence unaffected. Essentially, it is the study of molecular changes, including markers or mediators, as well as associated phenotypes that are mitotically or meiotically inheritable without changes in the DNA nucleotide sequence [14]. Epigenetic markers or mediators play a crucial role in biologically regulating gene expression. Thus, epigenomics is the study of epigenetic marks at a genome or near-genome scale [4]. Generally, epigenetic alterations occur in the tertiary structure of the DNA strands, which cause changes in the accessibility of DNA for molecules, thus affecting and regulating gene expression. Various factors, such as development in utero and in childhood, environmental chemicals, drugs and pharmaceuticals, aging, and diet, can influence these changes [3]. Although epigenetic inheritance goes against the idea that inheritance typically happens through the DNA code that passes from parent to offspring, epigenetic inheritance is gaining momentum as a heritable as well as reversible process at the cellular level [15]. The epigenetic changes are hereditary if the generation of the corresponding modifications occurs again on newly synthesized DNA or proteins of an organism’s offspring, which provides a hint that some forms are sustainably transmitted, but others are under conditions of erasing or resetting [16]. It is known that DNA methylation, histone modification, and noncoding RNA are three principal categories of epigenetic mediators [4] (Figure 1).

### 2.1. DNA Methylation

Epigenetic DNA modification is achieved by binding a methyl group from S-adenosyl-_L_-methionine to position 5 within the cytosine ring to produce 5-methyl-cytosine (5mC) [17]. The resulting function of DNA methylation is that gene transcription is repressed, in particular, DNA hypermethylation in the CpG islands (short sequences of genomic DNA with a higher frequency of the linear 5′-CpG-3′ sequence than other regions of the gene, where p stands for the phosphodiester bond connecting cytosine and guanine nucleotides) in the promoter region of genes, leading to gene silencing [3,18]. DNA methylation is considered to exert gene repression mainly through three mechanisms. Firstly, the methyl group could directly inhibit the DNA binding of some transcription factors, such as CTCF, NRF1, c-Myc, and HIF-1α [19,20]. Secondly, methyl groups could affect other epigenetic pathways like histone modifications. It has been demonstrated that DNA methylation represses the formation of the activating histone 3 lysine 4 (H3K4) di- and tri-methyl marks. Thirdly, methyl-binding proteins could bind specifically to methylated CpGs [21]. The members of this family, such as MBD1, MBD2, MBD3, MeCP2, and Kaiso, are able to bind repressor proteins and histone deacetylases (HDACs) [17,22]. Although, commonly, methylation of promoter CpG islands is associated with transcriptional repression, on some occasions, 5mC can act as a de-repressor through the displacement of the transcription silencing complex PRC2, leading to activation of transcription [23].

A family of DNA methyltransferases (DNMTs) have been identified and have demonstrated the ability to catalyze cytosine methylation in distinct sequence contexts. They include DNMT1, DNMT2, DNMT3a, DNMT3b, and DNMT3L. Notably, DNMT3L shows sequence homology with the DNMT3a/3b enzymes but lacks the very N-terminal region, including the PWWP domain [24]. Additionally, it also lacks a few essential catalytic motifs in the C-terminal region that include PC dipeptide at the active site as well as the sequence motif involved in binding the methyl donor S-adenosyl-L-methionine [24]. Thus, DNMT3L has no DNA methyltransferase activity [25,26,27]. Nevertheless, DNMT3L has been shown to interact with DNMT3a and DNMT3b and significantly stimulates their catalytic activities in vitro [28,29,30]. Furthermore, a study by the group Veland et al. uncovered the role of DNMT3L in maintaining DNMT3a stability, and, thus, DNMT3a-dependent DNA methylation [24].

Thus, in mammals, only DNMT1, DNMT2, and DNMT3a and 3b possess methyltransferase activity, while DNMT3L does not [31]. DNMT enzymes require S-adenosylmethionine (SAM) as an essential methyl donor cofactor, and, thus, metabolic perturbations are likely to influence 5mC profiles [32]. Interestingly, DNMT enzymes can be inhibited by xenobiotics and antisense or small interfering RNA, causing lower steady-state methyltransferase activity of global or gene-specific methylation [33]. Contrarily, in a study, it has been shown that exposure of human and rat aortic cells to a high-phosphate environment resulted in elevated DNMT activity and excessive methylation of the promoter region of SM22α [34]. Nonetheless, the activity of DNMT was decreased by adding the demethylating agent procaine to the high-phosphate medium since methylation of the SM22α promoter was prevented, which increased SM22alpha expression and decreased calcification [34].

The vast and main monogenic diseases resulting in arterial hypertension are related to renal sodium handling in the cortical collecting tubule (CCT) [35]. It is known that intracellular access of steroids to gluco- and mineralocorticoid receptors is regulated by reduced 11β-hydroxysteroid dehydrogenase enzymes 1 and 2 (11 β-HSD1 and 2) [36]. While 11β-HSD1 is predominantly expressed in the proximal collecting tubule (PCT), 11β-OHSD2 is in the CCT, and their corresponding oxidative activities declined in nephrectomised rats [36]. CpG islands that cover the promoter and exon 1 of 11 β-HSD2 are highly methylated in tissues and cell lines with low expression compared to those with high expression of 11 β-HSD2. Demethylation of 11 β-HSD2 enhances its transcriptional activity in human cells in vitro and in rats in vivo [37]. In contrast, methylation of 11 β-HSD2 promoter–luciferase constructs decreased transcriptional activity [37]. In addition, a methylated CpG-binding protein complex 1 transcriptional repression was found to interact with the methylated 11 β-HSD2. These findings suggested DNA methylation in 11b-HSD2 gene repression as an epigenetic mechanism causally linked with hypertension [37]. Similarly, methylation levels of CpG islands in the promoter of the endothelin-converting enzyme (ECE-1c) gene are also associated with blood pressure. It has been shown that methylation of these islands in vitro decreased the activity of the ECE-1c promoter [38].

### 2.2. Histone Modification

Histones are proteins forming octamers consisting of 2 H3-H4 and 2 H2A-H2B heterodimers, which are wrapped by 146 nucleotides of DNA to make a nucleosome. Nucleosomes are joined to each other via linker DNA to generate beads-on-the-string chromatin. Numerous post-translational modifications of histone proteins, such as lysine methylation and acetylation, are able to regulate transcription [39]. Histone acetylation is regulated by histone acetyltransferases (HATs) and histone deacetylases (HDACs), whereas histone lysine methylation is regulated by histone lysine methyltransferases and demethylases. During lysine acetylation, acetyl groups are transferred from acetyl-coenzyme A molecules to the lysine ε-amino groups of the histone tail. This reaction is catalyzed by three major families of HATs, namely GNAT, MYST, and CBP/p300. Nonetheless, the elimination of histone lysine acetylation is catalyzed by four families of HDACs, including class I (HDAC1–3, HDAC8), class II (HDAC4–7, HDAC9–10), class III sirtuins (SIRT1–7), and class IV (HDAC11). Both HATs and HDACs are recruited to target promoters in complexes of large multiproteins [40].

Epigenetic histone modifications are manifested as various post-translational changes (over 60 types) in the N-terminal tail (not the globular domain). Compared to methylation as the predominant mode of epigenetic DNA modification, diverse mechanisms are involved in modifying histone components, specifically, lysine modified by methylation, acetylation, ubiquitylation, and sumoylation, arginine modified solely by methylation, and serine and threonine modified by phosphorylation. As a result, the different patterns of histone modification offer distinct impacts on the transcriptional potential of the corresponding nucleosome. For instance, histone acetylation usually accelerates, while histone deacetylation represses, gene transcription; histone lysine methylation in position 79 restrains and histone arginine methylation actuates gene transcription; and histone monomethylation in position 9 activates, whereas hypermethylation in the same position suppresses, transcription [18,41]. More specifically, histone modifications are chemical modifications of the side chains of the H2A, H2B, H3, and H4 core, and there is substitution of the prototypical core histones by variant histones like H2AXZ or H2AZ. Histone modifications regulate gene expression since they determine chromatin conformation and control DNA accessibility of the components of gene transcriptional machinery, including RNA polymerases [42]. The number of histone modifications and their combinations encode the regulatory potential of the nearby genomic regions and are referred to as the “histone code” [43,44].

The central mechanism for gene regulation is the histone-dependent packing of genomic DNA into chromatin. The expression of inflammatory genes, DNA repair, and proliferation are determined by the degree of acetylation of histone and nonhistone proteins that are generated by histone acetyltransferases and HDACs [45]. Generally, histone deacetylases (HDACs) suppress gene expression. Class I HDACs, such as HDAC1, HDAC2, and HDAC3, primarily localizing to nuclei, are associated with epigenetics via their ability to efficiently deacetylate nucleosomal histones [46]. Class I HDACs are important in nephrogenesis. For instance, HDAC1–3 exhibit high expression in nephron precursors [47]. Histone deacetylase inhibitors (HDACi) have been applied to treat muscular diseases [48].

HDAC7 is a crucial factor in endothelial cell proliferation and growth as it prevents nuclear translocation of β-catenin and downregulates T cell factor-1 (TCF-1)/Id2 and cyclin D1, which results in G1 phase elongation. HDAC7 can be knocked down to cause β-catenin nuclear translocation and downregulate cyclin D1, cyclin E1, and E2F2, resulting in endothelial hypertrophy [49].

Cardiac hypertrophy has been related to cardiac dysfunction and heart failure. Activated HDAC2 results in hypertrophy via restraining the signal cascades of either Krüppel-like factor 4 (KLF4) or inositol polyphosphate-5-phosphatase f (Inpp5f) [50]. In a contrary study, class IIa HDACs have been proven to inhibit cardiac-specific transcription factors like myocyte enhancer factor 2 (MEF2) and GATA4 in the heart, leading to suppression of cardiac hypertrophy [51].

### 2.3. Noncoding RNA

Micro RNAs (miRNAs) are subsets of short noncoding RNAs of 21–26 nucleotides in length and are meditators of triggering tissue-specific post-transcriptional gene silencing [52]. They are short, single-stranded RNAs that are transcribed from noncoding genes and are formed in a two-step processing pathway mediated by two enzymes, Dicer and Drosha, in the category of RNAse III endonucleases. After 22 base sequences are processed, miRNAs connect to the same or similar target sequences in the 3′-UTRs of the gene, which prohibits translation or cleaving of the mRNA target [53].

Epigenetic regulation by noncoding RNAs is also executed by long intervening noncoding RNAs (lincRNAs) that employ chromatin-modifying complexes and/or overlay the coding region of genes, which, therefore, regulate gene expression at the level of specific target loci [54]. Furthermore, long noncoding RNAs (lncRNAs) show an ability to silence genes since they can recruit remodeling complexes, such as the polycomb complex, which is able to accelerate histone methylation. These RNAs also intend to recruit RNA-binding proteins that impair histone deacetylation or repress transcription factor binding to promoter regions [55].

Comparatively, methylation takes place through DNMTs, which silences genes. Histone acetylation and deacetylation are processes of post-translational modification, causing increased and decreased transcription of genes, respectively [56]. Nonetheless, noncoding RNAs work as post-transcriptional regulators, which function as guide molecules, pairing with partially or fully complementary motifs in 3′ untranslated regions (UTRs) of their target messenger RNAs (mRNAs) [57].

## 3. Epigenetic Cellular Modification and Hypertension

Generally, hypertension is a risk factor for a variety of vascular diseases that include atherosclerosis, restenosis, transplant vasculopathy, and vein graft failure. Atherosclerosis is defined as a change in the artery wall, variably featuring endothelial dysfunction, smooth muscle proliferation, extracellular matrix (ECM) accumulation, and lipid deposition [58]. It is caused by intimal thickening and hardening of the vessel wall with decreased elasticity and impaired blood circulation. The reduced vessel lumen size that is caused by atherosclerosis can result in complications like ischemia and hypertension [59].

Evidence has shown that epigenetics can be an important factor for cardiovascular diseases, including hypertension [60]. Epigenetic modifications in hypertension have been reported to change vascular biology, resulting in endothelial dysfunction, smooth muscle cell (SMC) proliferation, apoptosis resistance, and eventually remodeling of vasculature [61]. Therefore, it can be contemplated that hypertension is involved in various diseases, is associated with numerous physiological factors, and is epigenetically determined. To outline the relationship between hypertension and epigenetics, structural components of tissue are reviewed below on the basis of three principle epigenetic mechanisms.

### 3.1. Endothelial Cells

The endothelium plays a crucial role in vascular physiology, exhibiting diverse functions, such as regulation of vessel tone, angiogenesis, as well as immune cell adhesion and migration, exchange, and hemostasis [62]. Endothelial cells have constant exposure to circulating humoral factors, blood cellular constituents, other vessel wall types, and the physical forces of circulation because of their location along the blood vessel wall [63]. A connection has been found between arterial hypertension and endothelial dysfunction. Elevated blood pressure activates endothelium to cause endothelial dysfunction, leading to endothelial disintegration after a long period of offending stimulus. This eventually contributes to vascular rarefaction, which results in a decrease in tissue perfusion and consequent hypoxia, and a reduction in NO availability [8]. The promoter–reporter insertional transgenes of endothelial nitric oxide synthase (eNOS) are limited to expressing endothelial cells in the murine setting [64]. Histone modifications present at the eNOS proximal promoter enrich acetylation of histones H3 and H4 as well as methylation of lysine 4 of histone H3, which are associated with actively transcribed chromatin in endothelial cells [65].

Endothelial cells are also capable of expressing several proteins in vivo, making arterial and venous endothelial cells distinct. Some of these patterns are reserved in vitro, which implies that epigenetic mechanisms can affect long-term endothelial physiology [66]. Indeed, environmental factors can affect the progressive stages of forming atherosclerotic plaque via their interaction with the endothelium, which is the innermost lining of the vasculature system. This leads to endothelial dysfunction, disrupting its homeostatic functions [67]. Epigenetic mechanisms significantly affect the development of the vascular system. During the physiological changes, a primary capillary plexus is generated de novo from mesodermal cell precursors. By contrast, the generation of new vessels from pre-existing vessels is determined by the interaction of pro- and anti-angiogenic molecules. In the initial phase, vascular development is controlled by the presence of fibroblast growth factors for the induction of hemangioblastic differentiation. In the following phase, cell differentiation is triggered by vascular endothelial growth factor (VEGF). Forming a capillary plexus is largely based on the expression of adhesion molecules from intercellular connections, vascular endothelial (VE) cadherin, N-cadherin, and connexins, and molecules that promote cell–matrix interactions, such as netrins, semaphorins, fibronectin, integrins, and numerous signaling pathways including key proteins, such as NOTCH, VEGF1/2, transforming growth factor beta (TGF-β), or ephrin type-A receptor 2 (Eph-2) and 4 [68,69].

Additionally, epigenetic processes are able to effectively regulate the endothelial cell-restricted expression of eNOS. An independent study revealed that nine CpG dinucleotides in the promoter region of the eNOS gene in human endothelial cells presented low or no methylation [70].

#### 3.1.1. Effect of DNA Methylation on Endothelial Cells

The studies of DNA methylation have been related to eNOS, which is an endothelial-restricted factor with enzymatic activity for producing vasoprotective and vasodilatory endothelial nitric oxide (NO). Evidence indicates that increasing the DNA methylation level of endothelial-specific genes directs the reprogramming of mature human endothelial cells toward an embryonic stem cell-like phenotype [71], while inducing DNA demethylation of GATA-2, GATA-3, and eNOS promoters in cultured human embryonic stem cells triggers differentiation toward mature endothelial cells [72].

**Figure 2:**
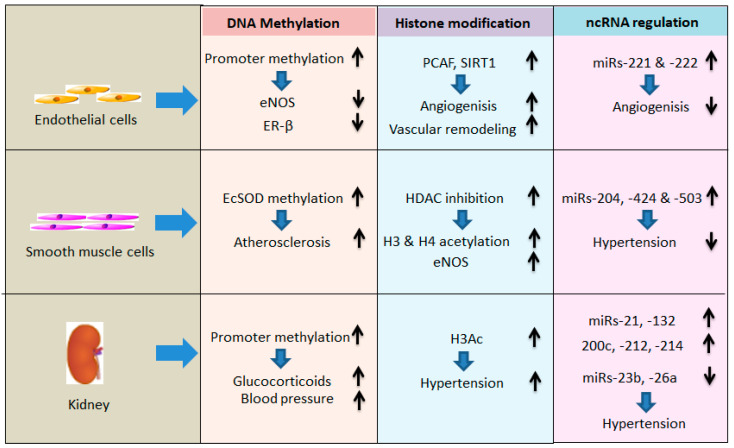
Epigenetic cellular modification and hypertension. Abbreviations: eNOS, endothelial nitric oxide synthase; ER-β, estrogen receptor-β; PCAF, P300/CBP-associated factor; SIRT1, Sirtuin 1; EcSOD, extracellular superoxide dismutase; HDAC, histone deacetylase; H3Ac, acetylated histone H3.

Human endothelial cells from atherosclerotic plaques exhibit increased DNA methylation at the promoter region and decreased levels of estrogen receptor-β (ER-β) of the gene compared with those from non-atherosclerotic plaques [73]. Lacking lysine-specific demethylase-1 (LSD1, KDM1a) has been related to decreased expression of eNOS, NO-dependent vasodilation, and salt-sensitive hypertension [74]. In brief, increased DNA promoter methylation decreases the expression of eNOS and ER-β (Figure 2).

#### 3.1.2. Effect of Histone Modifications on Endothelial Cells

Evidence has indicated that HATs are able to catalyze intragenic histone acetylation, and are also a crucial factor in vessel formation. A member of HATs, PCAF, is responsible for arteriogenesis, which is a remodeling of pre-existing collateral arterioles into larger arteries [75]. Mice lacking HBO1, which is a prominent H3K14 HAT, are embryonically lethal, demonstrating embryonic vascular remodeling [76]. Recent findings also show that *SIRT1*, a member of the HDAC gene family, contributes to angiogenic signaling. SIRT1 manages the angiogenic activity of endothelial cells, which is expressed in the vasculature during blood vessel growth. Deficiency of *SIRT1* function retards the sprouting angiogenesis and branching morphogenesis of endothelial cells, causing downregulation of genes for blood vessel development and vascular remodeling [77] (Figure 2).

#### 3.1.3. Effect of Noncoding RNA Regulation on Endothelial Cells

miRNAs are essential for regulating angiogenesis. Adult endothelial cells with silenced *Dicer* or, to a lesser extent, *Drosha* led to the downregulation of positive regulators of the angiogenic phenotype and impaired tube generation on Matrigel [78]. Lacking miR17-5p and let7b miRNAs that downregulate the expression of the anti-angiogenic TIMP1 has been linked to damage in angiogenesis in Dicer-deficient mice [79]. miR-221 and -222 repress angiogenic processes through tube formation and endothelial cell migration [80] (Figure 2).

### 3.2. Smooth Muscle Cells

Smooth muscle cells (SMCs) are an unusual cell type with remarkable plasticity, which are able to readily change between two phenotypic states, contractile and synthetic, based on environmental stimuli. Vascular smooth muscle cells (VSMCs) are an essential component in forming the artery structure, offering the necessary contractile function to maintain blood flow. Under activation, SMC proliferation and migration occur into the intima, which ultimately causes the generation of an atherosclerotic plaque [81]. The SMCs within adult blood vessels present the contractile phenotype, which features low proliferation rates, a high extent of cytoplasmic myofilaments, low rates of protein synthesis, and a unique repertoire of contractile proteins, such as smooth muscle α-actin (ACTA2), smoothelin, h-caldesmon, calponin, transgelin (TALGN), and smooth muscle myosin heavy chain (MYH11) [82].

#### 3.2.1. Effect of DNA Methylation on SMCs

DNA methylation has been reported to regulate certain SMC genes, resulting in the modulation of the SMC phenotype and the development of vascular diseases. Hypermethylation of the extracellular superoxide dismutase gene (EcSOD) has been related to the formation of atherosclerosis [83,84] (Figure 2). Phenotypic modulation of SMCs can cause vascular calcification. 5-Aza-2′-deoxycytidine can inhibit DNMTs to promote the mineralization of cultured human aortic SMCs, likely via demethylation of the alkaline phosphatase promoter [85]. Treating VSMCs with 5-azacytidine, which is an inhibitor of DNA methyltransferase, increases eNOS mRNA in these cells [86].

#### 3.2.2. Effect of Histone Modifications on SMCs

An oxidized phospholipid, 1-palmytoyl-2-(5-oxovaleroyl)-sn-glycero-3-phosphocholine (POVPC), is found within atherosclerotic lesions and related to monocyte infiltration. POVPC elevates the presence of HDAC2 and HDAC5 at the *ACAT2* and *TAGLN* promoters in a KLF4-dependent manner, which contributes to the hypoacetylation of histone H4 [87]. Ang II is a mediator of vascular remodeling during hypertension, governing SMC hypertrophy through regulating HDAC activity. SMCs treated with Ang II are found to trigger phosphorylation of HDAC5 at Serine 259/498 in both time- and dose-dependent manners. Likewise, Ang II treatment also causes phosphorylation via Calmodulin kinase II and nuclear export of HDAC4, which leads to the reduction in myocyte enhancer factor-2 (MEF2), which is a hypertrophic transcription factor [88,89]. The treatment of an HDAC inhibitor, trichostatin A (TSA), on spontaneously hypertensive rats drops blood pressure and vascular inflammation [90]. Likewise, VSMCs under treatment with TSA increase eNOS mRNA in these cells and raise H3 and H4 acetylation at the eNOS proximal promoter (Figure 2). Evidence has also shown that MeCP2, HDAC-1, and HDAC-2 are basally associated with the eNOS promoter in VSMCs [86].

#### 3.2.3. Effect of Noncoding RNA Regulation on SMCs

It has been shown that inhibition of miR-145 decreases vascular remodeling and right ventricular dysfunction in the hypoxia model of pulmonary arterial hypertension [91]. Downregulation of miR-204 in pulmonary hypertension has been linked with disease severity and SMC proliferation. Delivery of synthetic miR-204 can lessen disease in an animal model of pulmonary hypertension [92]. Similar analysis also indicates that restoration of miR-424 and miR-503 expression mitigates pulmonary hypertension (Figure 2) in animal models by involving mechanisms regulating endothelial modulation of SMC proliferation [93]. In addition, evidence has also indicated that miR-143, miR-145, miR-21, miR-133, and miR-1 were associated with VSMCs’ phenotypic modulation and AH [94].

### 3.3. Kidney

It is well known that the adult kidney consists of a large number of specialized epithelial, podocyte, and mesangial cells, among others. Epigenetic studies in kidney disease are still in the preliminary stage despite evidence that has shown epigenetic differences are associated with chronic kidney disease (CKD) development. Since the epigenome is cell type-specific, the elucidation of results from mixed cell types in organs, such as kidneys, remains difficult [95] and requires analysis at single-cell resolution [96]. Nonetheless, three principal epigenetic mechanisms, i.e., DNA methylation, histone modification, and noncoding RNA regulation, are discussed below with a special emphasis on kidney cell types: podocytes, mesangial, and epithelial cells. Since there is not much information related to hypertension alone, here we discuss it in the context of other kidney diseases, including diabetic nephropathy (DN), which is often accompanied by hypertension.

#### 3.3.1. Effect of DNA Methylation on Podocyte, Mesangial, and Epithelial Cells

A study was carried out to profile genome-wide cytosine methylation in tubule epithelial cells obtained from CKD and control kidneys. The result showed that 4751 differentially methylated regions (DMRs) were identified in 26 diabetic and hypertensive CKD samples based on the comparison with control samples [97]. Exposure to excess glucocorticoids in the fetal stage results in hypertension development in adult offspring. Renal expression of the glucocorticoid receptor in 6-month-old offspring is elevated several times after maternal treatment with dexamethasone. The results from the methylation-sensitive polymerase chain reaction (PCR) demonstrate that this upregulation of the renal glucocorticoid receptor is related to hypomethylation of the glucocorticoid receptor promoter [98] (Figure 2). Maternal exposure to a low-protein diet causes hypertension in adult offspring, which is found to result in upregulation of the angiotensin type 1 receptor and hypomethylation of its promoter in the adrenal gland [99]. The 11β-hydroxysteroid dehydrogenase enzyme (11β-HSD2) shows a sound cell-specific constitutive expression in mineralocorticoid target tissues like epithelial cells from the renal cortical collecting duct. Methylation of the 11β-HSD2 promoter contributes to decreased transcriptional activity. The expression of the reduced binding activity of this enzyme is controlled by methylation of recognition sequences of transcription factors, suggesting that DNA methylation for the expression of this gene is causally linked with hypertension [37]. Corroborating these findings, we have also shown that methylation-dependent (mediated by DNMT1, 3a, and 2b) regulation of genes involved in antioxidant redox balance, especially MnSOD, CuSOD, Nox4, and SIRT1, are involved in hypertensive kidney injury in aging [100].

In a study using human samples, Hayashi et al. reported that DNA methylation and DNA damage in podocytes are potential markers for kidney function decline in IgA nephropathy. However, they recognized that these candidate markers have not been adequately investigated in other glomerular diseases [101]. To look into other glomerular diseases, the same group led by Yoshimoto N. reported that podocyte damage as well as glomerular DNA methylation were associated with the severity of proteinuria in KCD patients that include minor glomerular abnormality, membranous nephropathy, and diabetic nephropathy [102,103]. Using an in vitro study, Zhang et al. demonstrated that levels of podocyte slit diaphragm proteins, nephrin, and podocin DNA methylation caused increased podocyte injury in hyperglycemia conditions. Interestingly, they also reported that DNA methylation inhibitor decreased hypermethylation of these podocyte slit diaphragm genes both in vivo and in vitro [104]. Additionally, using a widely used clinical anticancer drug Mithramycin A, which has demethylation properties, in animal models, they have shown that it mitigated the urinary excretion of albumin, glomerular mesangial expansion, and podocyte injury and improved kidney function in DN, suggesting that DNA hypermethylation causes podocyte injury and renal function decline [104]. In a recent review, Hayashi et al. nicely summarized the available literature, including their breakthrough finding using both animal and human specimens regarding the role of altered DNA methylation in glomerular podocytes, focusing on transcription factors and DNA damage that are related to decline in renal function [105].

The epithelial sodium channel (ENaC) is expressed in the apical membrane of the epithelia of the kidney and has a major role in Na^+^ reabsorption in the distal tubule, and hence the regulation of Na^+^ balance, extracellular fluid volume, and blood pressure [106,107]. A CpG island near the transcription start site of the αENaC promoter is regulated by the control of promoter methylation status [108], and a report suggested that 5-Aza-2′-deoxycytidine-mediated promoter demethylation enhanced Sp1 binding to, and transactivation of, the αENaC promoter, and thus increased αENaC mRNA expression in cells exhibiting epithelial morphology that were isolated from the kidney of adult mice [109]. These results suggest that DNA methylation/demethylation in epithelial cells plays a pivotal role in regulating blood pressure.

#### 3.3.2. Effect of Histone Modifications on Podocyte, Mesangial, and Epithelial Cells

Epigenetic regulation by histone deacetylases (HDACs) and alterations of the renin–angiotensin system (RAS) are involved in the developmental programming of hypertension [110]. It is reported that melatonin restrains programmed hypertension resulting from neonatal dexamethasone (DEX) exposure that increases HDAC 1–3 protein levels and inactivates most renin–angiotensin system (RAS) genes in the kidney [110]. This result was from the whole rat kidney. Thus, it was unknown whether this effect was partly from podocyte, mesangial, or epithelial cells. In an independent study, the result shows that acetylated histone H3(H3Ac) and di-methylated lysine 4(H3K4me2), activating histone codes, are around 25- and 3-fold higher in the kidneys of spontaneously hypertensive rats (SHR) than in Wistar-Kyoto (WKY) rats, respectively (Figure 2). However, di-methylated histone H3 at lysine 9 (H3K9me2), a suppressive histone code, exhibits 50-fold lower in SHRs than in WKY rats. These findings implicate that the kidneys of SHRs contain more activating histone codes [111]. Evidence also demonstrates that the HDAC inhibitor, suberoylanilide hydroxamic acid (SAHA), lessens the cardiovascular remodeling linked to DOCA-salt hypertensive rats, improving cardiovascular structure and function, in particular fibrosis, in the heart and blood vessels, possibly by restraining inflammation [112].

Podocyte injury contributes to the loss of integrity of the glomerular filtration barrier, which results in proteinuria. It is reported that germline loss of murine podocyte-associated HDAC1 and HDAC2 results in proteinuria and collapsing glomerulopathy due to sustained double-stranded DNA damage [113]. Similar findings reported that *HDAC1* and *HDAC2* genes contributed to the pathogenesis of proteinuria in murine and human ESRD [114], and histone H3K4 methylation in glomerular podocytes associated with proteinuria in patients with membranous nephropathy [115]. Another study investigated the role of HDACs in renal injury induced by hyperhomocysteinemia, a non-protein amino acid at its higher level, that also contributes to the rise in blood pressure [116]. The study identified the expression patterns of HDACs, particularly zinc-dependent HDAC9, were preferentially upregulated in the mouse kidney with HHcy. They also found that deficiency or pharmacological inhibition of HDAC9 ameliorated renal injury, and podocyte-specific deletion of HDAC9 significantly attenuated podocyte injury and proteinuria in these mice [116]. The results further suggested that HDAC9 reduced the acetylation level of histone H3 at lysine 9 (H3K9) in the promoter of Klotho, then inhibited the gene transcription of Klotho, finally aggravating podocyte injury in HHcy [116]. These findings, along with other studies, suggest that HDACs are major regulators of podocyte injury and proteinuria [117].

In an in vitro study using rat mesangial cells, Reddy et al. showed that expression levels of RAGE, PAI-1, and MCP-1 mRNAs as well as H3K9/14Ac at their promoters were increased following high glucose induction that mimics in vivo diabetic conditions [118]. They further confirmed these with ChIP assays and reported that increased H3K9/14Ac at the RAGE, PAI-1, and MCP-1 promoters was further augmented by high glucose with angiotensin treatment [118]. Furthermore, acetylation of these promoters was ameliorated by losartan treatment, suggesting that losartan inhibits histone post-translation modification in diabetic conditions [118].

Hyndman et al. reported kidney medulla-specific inhibition of class I HDACs in rats with high-salt feed-induced hypertension and fluid–electrolyte imbalance [119]. With additional inducible murine models, they were able to demonstrate that HDAC1 and HDAC2 in the kidney epithelium are necessary for maintaining epithelial integrity and fluid–electrolyte balance during increased dietary sodium intake [119]. These results suggest that kidney tubular epithelial HDACs are necessary for fluid–electrolyte balance with high-salt diets, which otherwise lead to hypertension and kidney damage [119].

#### 3.3.3. Effect of Noncoding RNA Regulation on Podocyte, Mesangial, and Epithelial Cells

An early study demonstrated that angiotensinogen (AGT), the primary substrate of the renin–angiotensin–aldosterone system (RAAS), was able to affect miR-21 expression, and its overexpression resulted in a rise in aldosterone secretion and cell proliferation, implicating the changes in miR-21 expression levels causing primary aldosteronism and hypertension [120]. In a study on salt-induced arterial hypertension (AH) in a rodent model, an increase in miR-320 and miR-26b and a reduction in miR-21 were seen. The insulin growth factor-1 receptor (*IGF1R*) was considered a putative target of miR-320, whereas phosphatase and tensin homolog (*PTEN*) on chromosome 10 was described as a putative target of miR-26b and miR-21. Downregulation of *IGF1R* and upregulation of *PTEN* in the rat aortas were deemed the reason for a high-salt diet causing vascular remodeling and increased fibrosis [121].

A study has been conducted to analyze miRNAs in kidneys related to hypertension. The result showed that 12 genes and 11 miRNAs were differentially expressed in the renal medulla, while 46 genes and 13 miRNAs were in the cortex. Moreover, the differentially expressed miRNAs and genes were further compared in hypertensives and normotensives. Based on the qPCR results, miRNAs hsa-miR-638 and hsa-let-7c were validated in the renal medulla samples, whereas hsa-miR-21, hsa-miR-126, hsa-miR-181a, hsa-miR-196a, hsa-miR-451, hsa-miR-638, and hsa-miR-663 were confirmed in the cortex samples. These results hinted that the alteration in these miRNAs is involved in the overexpression of renin mRNA observed in the hypertensive kidneys [122]. In a study regarding RAS blood pressure, miR-132 and miR-212 were upregulated in the kidneys, heart, and aorta in the Ang II-treated rats, suggesting it may mediate Ang II-induced hypertension [123] (Figure 2).

A study by Ursu et al. reported that miR-200c was upregulated in urine from patients with podocytopathies [124]. Their group also found that miR-200c overexpression caused proteinuria, edema, podocyte foot process effacement, and glomerular endotheliosis in Zebrafish. They further proved that miR-200c decreased VEGF-A expression and secretion in cultured human podocytes, suggesting that miR-200c can cause glomerular damage, likely due to the reduction in podocyte VEGF-A [124]. Another study found that miR-26a, a major regulator of TGF-β signaling, was downregulated in exosomes isolated from urine and as well as from the plasma of hypertensive normo-albuminuria patients compared with healthy controls [125]. Similarly, decreased miR-26a levels were found in podocyte-derived exosomes after TGF-β stress. These results revealed that miRs, particularly miR-26a, are key molecules associated with albuminuria in hypertension [125] (Figure 2).

In a human study, Li et al. showed that miR-23b levels are downregulated in kidney biopsies and serum of patients with IgA nephropathy [126]. They further showed 23b−/− mice developed an IgA nephropathy-like phenotype of mesangial IgA and C3 deposition associated with the development of albuminuria, hypertension, and elevated serum creatinine, suggesting that loss of miR-23b is indispensably associated with IgA nephropathy [126] (Figure 2).

Denby et al. examined the role of TGF-β-induced expression of miRNAs in cell culture and models of renal diseases [127]. Their study revealed that TGF-β changed the expression of miR-21, miR-214, and miR-145 in rat mesangial cells and miR-214, miR-21, miR-30c, miR-200b, and miR-200c during induction of epithelial–mesenchymal transition in rat tubular epithelial cells, with a robust increase in miR-214 expression in both cell types. In contrast, in tubular epithelial cells, miR-21 was increased, and miR-200b and miR-200c were decreased in response to TGF-β [127]. To further assess the differential expression of these miRNAs in vivo, they used three different models, of mesangial glomerulonephritis, unilateral ureteral obstruction, and stroke-prone spontaneously hypertensive rats. Their findings revealed that the expressions of miR-214 and miR-21 were significantly increased in all in vivo models (Figure 2), suggesting miRNAs play a pivotal role in renal damage in differing diseases, including hypertension [127].

## 4. Hypertension-Related Kidney Damages

It is well known that hypertension and renal disease are highly associated. Historically, renal damage triggered by hypertension can be divided into two differing clinical and histological patterns, regarding “benign” and “malignant” nephrosclerosis [8]. The degree of kidney damage caused by hypertension is proportional to the extent of the arterial pressure exposure of the renal microvasculature. When the arterial pressure increases severely to exceed the range of autoregulatory protection, it causes the transmission of a modest and transient arterial pressure increase to the glomerular capillaries, resulting in acute disruptive vascular and glomerular injuries with fibrinoid necrosis in afferent arterioles, i.e., malignant nephrosclerosis [8].

### 4.1. Nephron Loss

**Figure 3:**
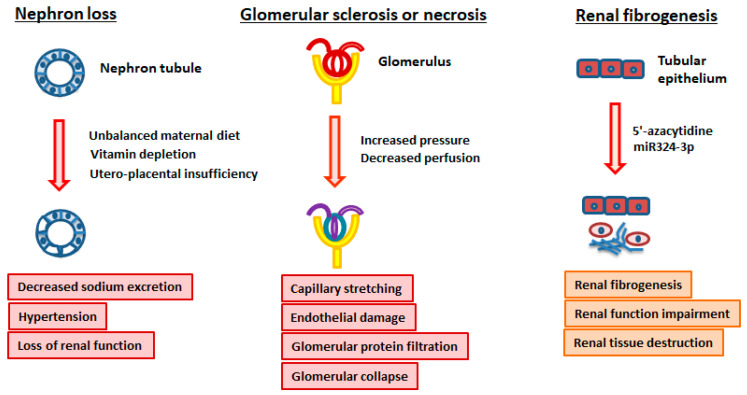
Hypertension-related kidney damages.

The nephron, the functional unit of the kidney, is in charge of purifying and filtering the blood, and contains a glomerulus, the proximal and distal convoluted tubules, Henle’s loop, and the collecting duct. It has been increasingly reported that epigenetic changes may be a crucial factor in the pathogenesis of kidney diseases. Permanent structural and functional effects on the developing kidney can be caused by certain environmental stimuli during the critical period of kidney organogenesis in utero. For example, an inborn deficit in the number of nephrons in the developing kidney can result from an unbalanced maternal diet, vitamin depletion, or utero-placental insufficiency [128]. This nephron loss further causes a decrease in sodium excretion and provokes an increased risk of developing hypertension in adulthood (Figure 3). Additionally, functioning renal loss can occur with compensatory hypertrophy and hyperfiltration in the remaining nephrons, falling into the vicious cycle of nephron loss and raising susceptibility to future renal injury and hastened loss of renal function [128] (Figure 3). These nutritional and stress factors that take place during the early phases of development lead to changes in the epigenetic regulation of embryonic gene expression and influence the whole process of fetal programming [129].

### 4.2. Glomerular Sclerosis or Necrosis

The glomerulus is a specialized filtration tissue in the renal cortex, which controls the passage of macromolecules from the bloodstream into the urinary space. Three types of cells constitute this complex structure, including podocytes, which are highly specialized visceral epithelial cells, mesangial cells that are modified SMCs, and endothelial cells [130]. Blood is filtered by passing through glomeruli, which allows filtered water and metabolic wastes to pass into the lumen of the Bowman’s capsule and drain into the tubules, where reabsorption takes place. In kidney diseases, glomeruli are the site of injury [131,132].

Essentially, glomerular hypertension causes glomerular capillary stretching, endothelial damage, and elevated glomerular protein filtration, which leads to the collapse of glomeruli, segmental necrosis, and glomerulosclerosis. Glomerular collapse is due to decreased perfusion, while increased pressure triggers either glomerular sclerosis or necrosis (Figure 3). In addition, sclerosis of preglomerular vessels further drops renal blood flow (RBF) [8].

A study indicated that the morbidity and mortality of *Chd2*^+/mut^ mice were related to significant kidney disease influencing the glomerulus and tubules. Changes were especially found in the glomerulus, exhibiting elevated thickening of the basement membrane, such as an increased deposition of collagen. The result suggested the ability of the ChD2 protein to maintain kidney function. Basically, the ChD2 protein is a member of enzymes that are involved in ATP-dependent chromatin remodeling, which can be applied as an epigenetic regulator of gene expression through modification of chromatin structure [133].

### 4.3. Renal Fibrogenesis

Renal fibrogenesis is a non-self-limited pathological repair process, persisting independently from the initial stimuli, which results in renal tissue destruction and renal function impairment [134]. Those fibroblasts displaying elevated proliferation and increased synthesis of ECM components are the major mediators of the renal fibrotic process [135]. It is demonstrated that 5′-azacytidine, a demethylating agent, improves experimental kidney fibrosis in mice and diminishes the phenotypic activation of fibrotic fibroblasts in vitro (Figure 3). Moreover, the treatment with the profibrotic factor transforming growth factor beta-1 (TGF-β1) has an effect on DNA methyltransferase 1 activity and, consequently, the DNA methylation status [136].

It has been demonstrated that adequate glycemic control has a protective ability against kidney injury in diabetic patients [137]. Also, transient hyperglycemia triggers enduring epigenetic modifications in the promoter of the nuclear factor kB (NF-kB) subunit p65 in aortic endothelial cells [138].

Epigenetic modifications have an impact on hypertensive nephropathy. It has been shown that in a rat model of spontaneous progressive nephropathy, upregulation of miR324-3p has an association with renal fibrosis (Figure 3). However, this miR324-3p overexpression can be turned down by angiotensin-converting enzyme (ACEI) [139].

## 5. Metabolic Disorders, Hyperhomocysteinemia, and Hypertension

Homocysteine (Hcy) is a sulfur-containing nonessential amino acid derived from the intermediate metabolites of methionine. High plasma Hcy levels, also known as hyperhomocysteinemia (HHcy), are regarded as a common feature of uremia in chronic renal failure patients [140]. It has been revealed that HHcy causes elevated levels of its precursor, S-adenosylhomocysteine (SAH), which performs as a powerful competitive inhibitor of S-adenosylmethionine (SAM) in DNA methylation reactions. In this process, SAM acts as a methyl group donator for various acceptors [141]. HHcy also leads to a reduction in the activity of DNA methyltransferase 1 and demethylation of the promoter region of cyclin A in endothelial cells, which blocks cell cycle progression and endothelium regeneration [142].

**Figure 4:**
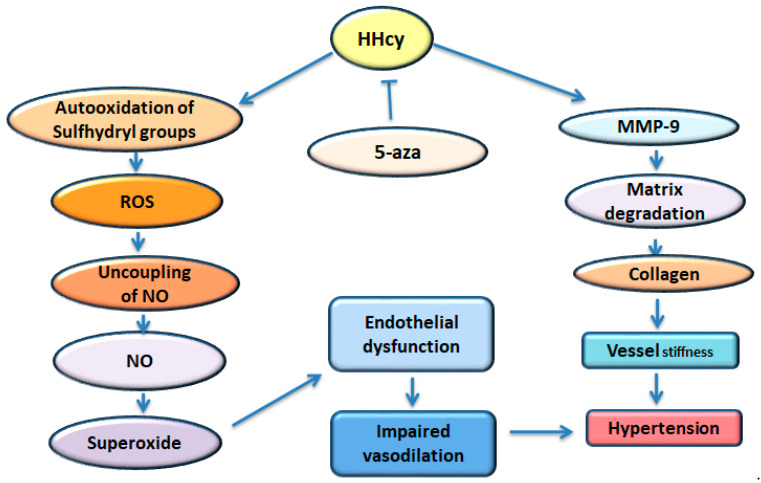
Metabolic disorders, hyperhomocysteinemia, and hypertension. HHcy: hyperhomocysteinemia, ROS: reactive oxygen species, NO: nitric oxide, 5-aza: 5-aza-2′-deoxycytidine, MMP-9: metalloproteinase-9.

During this physiological evolution, HHcy, being an initiator, also plays a vital role as it leads to the auto-oxidation of sulfhydryl groups, which accelerates the production of reactive oxygen species (ROS), further uncoupling endothelial nitric oxide (NO) reactions to decrease NO synthesis and bioavailability [143]. The reduced NO signaling elevates superoxide generation to produce a vicious cycle, impairing endothelial function. The resulting endothelial dysfunction impairs vasodilation to induce arterial hypertension [144] (Figure 4).

Vascular pathology is associated with HHcy, which is featured by the developing excess ECM. This process increases the deposition of collagen, causing vessel stiffness [145]. In fact, HHcy raises the expression and activity of metalloproteinase-9 (MMP-9), contributing to matrix degradation and accumulation of collagen in the ECM [146].

Our previous studies demonstrated that under the context of HHcy, cystathionine-γ-lyase (CSE) could be activated by Hcy with homocysteinylation, which occurs in hypertension and other cardiovascular diseases [147].

As one of the sound therapies against hypertension, the epigenetic approach has exhibited potential and promising prospects. The result from one of our previous studies indicated that the treatment of Aza [5-aza-2′-deoxycytidine, a DNA methyltransferase (DNMT1) inhibitor] in HHcy mice dropped DNA methylation and thus decreased adverse aortic remodeling to alleviate hypertension [148] (Figure 4). Practically, after Aza application, some favorable alterations were observed, such as a normalization of the plasma Hcy level and blood pressure, a decrease in the resistive index and wall-to-lumen ratio, a reduction in collagen deposition in the aorta, and an improvement in vascular response to phenylephrine, acetylcholine, and sodium nitroprusside. Aza treatment reduced the expression of DNA methyltransferase 1 (DNMT1), MMP-9, metalloproteinase 1 (TIMP1), and *S*-adenosyl homocysteine hydrolase (SAHH) and upregulated methylene tetrahydrofolate reductase (MTHFR) [148]. The cytokine interferon-γ (INF-γ) inhibits expression of the COL1A2 gene in SMC, resulting in destabilization of atherosclerotic plaques. This effect has been seen via INF-γ-dependent recruitment of repressor complex, including HDAC2 and the H3K9 methyltransferase G9a [149].

## 6. Protein Homocysteinylation and Hypertension

**Figure 5:**
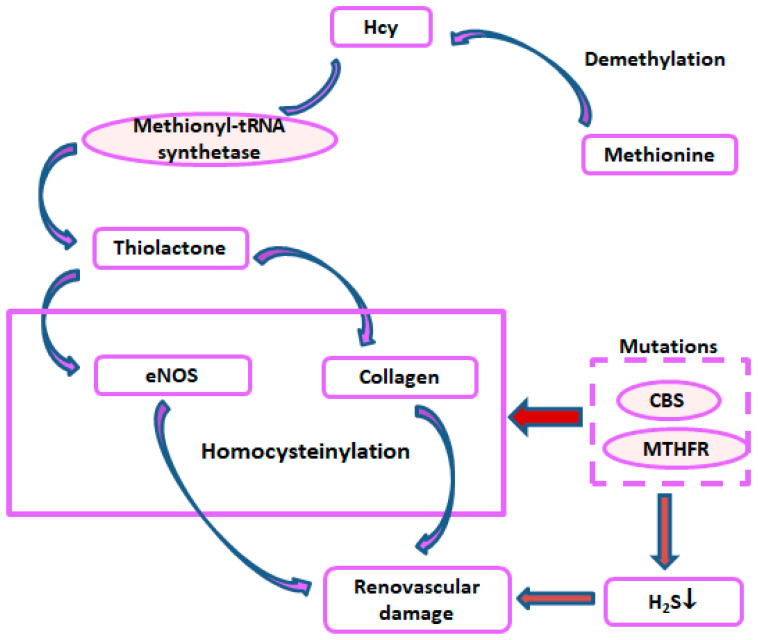
Protein homocysteinylation and hypertension. Hcy: homocysteine, eNOS: endothelial nitric oxide synthase, CBS: cystathionine β-synthase, MTHFR: methylenetetrahydrofolate reductase.

Homocysteine (Hcy) is a non-protein-coding amino acid synthesized by the demethylation of methionine (Figure 5). The methyl group, which is excised during the synthesis of Hcy, is used in various methylation reactions involving DNA, proteins, amino acids, and Hcy [150]. Contrarily, at an elevated level, Hcy inactivates proteins by homocysteinylation [151].

In fact, H. Jakubowski and his group have extensively worked on protein homocysteinylation and its effects on various pathological consequences [152]. In the late nineties, by incubating human serum with Hcy-thiolactone, they showed that protein homocysteinylation was a major reaction, which was proportional to their lysine residues [153]. Particularly, four lysine residues (Lys8 or -13, Lys86 or -87, Lys99, and Lys100) of cytochrome c are detected to be susceptible to N-homocysteinylation, resulting in a redox imbalance of the heme ligand of cytochrome c [154]. This homocysteinylation perhaps led to protein damage, an indication that may underlie HHcy in vascular diseases [153]. Interestingly, a year later, they also showed that the detoxification of Hcy-thiolactone is protective against protein homocysteinylation, which otherwise would potentially contribute to atherosclerosis [155]. To add to these groundbreaking discoveries, further studies indicated that an enzyme, methionyl-tRNA synthetase, effectively converted Hcy to thiolactone (Figure 5), and it was directly proportional to Hcy concentration and inversely proportional to methionine concentration [156]. This finding further suggested that the metabolic conversion of Hcy to thiolactone and protein homocysteinylation by thiolactone may be involved in Hcy-induced vascular damage [156].

Further to these above studies, it has also been shown that protein N-homocysteinylation involves a post-translational modification by Hcy-thiolactone. A major portion of circulating Hcy was found to be N-linked to hemoglobin and albumin, and Lys(525) was a predominant site of N-homocysteinylation in human serum albumin in vitro as well as in vivo [157]. It was also shown that protein N-homocysteinylation affects the susceptibility of albumin to oxidation and proteolysis [157]. Interestingly, antibodies against Nepsilon-homocysteinylated albumin were shown to be associated with early-onset coronary artery disease, an indication of an autoimmune response to Nepsilon-Hcy-albumin that may represent a new mechanism involved in the early development of CAD [158]. It has also been suggested that the Hcy-related autoimmune response is resistant to folic acid administration in CAD patients [159], which might explain in part the failure of vitamin therapy to reduce the risk of cardiovascular events, as reported [160,161].

Later it was discovered that mutations in the cystathionine beta-synthase (CBS) or methylenetetrahydrofolate reductase (MTHFR) gene, which are involved in Hcy metabolism, increase N-homocysteinylated protein levels in humans, which could explain why increased atherothrombosis is observed in CBS-deficient patients [162]. Additionally, N-homocysteinylation of collagen in CBS-deficient mice has been shown to impair its cross-linking [163]. Collagen is an important structural protein in connective tissue that maintains vascular integrity, and the N-homocysteinylation of collagen suggests it may impact BP in HHcy patients. In fact, we have shown that the homocysteinylation of another BP-regulating enzyme, i.e., eNOS, in addition to causing a reduced production of H_2_S, an anti-hypertensive agent, resulted in contributing to the increase in BP in CBS+/− mice [151]. These double-edged effects of HHcy and reduced H_2_S production exemplified hypertension and renovascular damages [151] (Figure 5).

Genetic or nutritional deficiencies in Hcy metabolism increase Hcy-thiolactone, which causes protein damage by forming isopeptide bonds with lysine residues, generating N-Hcy-protein. A work authored by Boroczyk et. al. showed the prevalence and genetic determinants of keratin damage caused by homocysteinylation in mammals and birds. They showed that a large amount of Hcy was bound to hair keratin via amide or isopeptide bonds (Hcy-keratin), while a relatively low amount was found as S-Hcy-keratin [164]. Since the homocysteinylation was associated with pelage, it was unknown from this study whether keratin homocysteinylation was associated with blood pressure regulation. Interestingly, Keratin 1 has been shown to attenuate hypoxic pulmonary artery hypertension by inhibiting arterial smooth muscle expansion [165]. Thus, it is possible that Keratin 1 hypermethylation may cause a rise in arterial blood pressure as it inactivates protein. However, this possibility needs to be explored and validated with experimental evidence.

## 7. Oxidative Stress

There is a strong relationship between hypertension and oxidative stress. Activation of ROS-forming enzymes in endothelial and VSMCs produces redox signaling to stimulate inflammation transcription factors. ROS can potently modulate vascular contraction and dilation, reducing nitric oxide bioavailability, which causes lipid peroxidation, activating pro-inflammatory transcription factors, increasing growth factor production, and inducing fibrosis. ROS are able to strengthen afferent arteriolar tone and reactivity indirectly through potentiation of tubuloglomerular feedback, or directly via alleviating endothelium-derived relaxing factor or NO response [166].

Overproduction of reactive oxygen species can be induced by transient ischemia and reperfusion. A significant alteration was found in the ratio of methylated to unmethylated CpG islands within the interferon-gamma response element in the promoter region of the complement 3 (C3) gene in the rat kidney after going through 24 h of cold ischemia followed by 2 h of warm reperfusion [167].

## 8. Contribution of Biologically Active Gases in Epigenetic Hypertension

**Figure 6:**
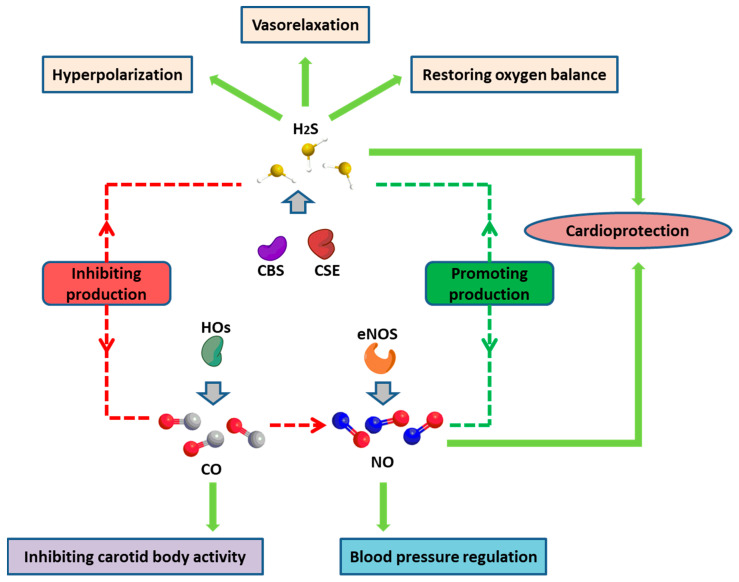
Main functions of biologically active gases, NO, CO, and H_2_S, and their interaction. CBS, cystathionine-β-synthase; CSE, cystathionine-γ-lyase; HOs, heme oxygenases.

Biologically active gases including nitric oxide (NO), carbon monoxide (CO), and hydrogen sulfide (H_2_S) are crucial for performing overlapping homeostatic functions in the renovascular system [168]. As shown in Figure 6, those gases not only play a crucial role in regulating the function of endothelial cells but also affect each other to maintain the balance of physiological conditions.

### 8.1. Hydrogen Sulfide (H_2_S)

Three enzymatic systems have been found to form H_2_S in mammals, namely cystathionine-β-synthase (CBS), cystathionine-γ-lyase (CSE), and 3-mercaptopyruvate sulfurtransferase (MPST). H_2_S is able to cause endothelium-dependent vasorelaxation, which is an endothelium-derived hyperpolarizing factor [169]. For example, losing endothelium diminishes the relaxation of rat aortic tissues triggered by H_2_S [170]. H_2_S-induced vasorelaxation depends on the activation of the ATP-sensitive K^+^ channel (K_ATP_) in vascular smooth muscle by raising whole-cell K_ATP_ currents to hyperpolarize membrane potentials and enhancing single-channel activity via increasing the permeability of single K_ATP_ channels [171].

Under physiological conditions, the renal medulla is a highly hypoxic environment. The renal medulla has a higher content of H_2_S than the renal cortex even though the expression of H_2_S-synthesizing enzymes is low since mitochondrial H_2_S oxidation is slow. Careful regulation of the renal medullary oxygen balance is required as any further reduction in pO_2_ leads to damage to tubular cells. H_2_S acts as a crucial oxygen sensor in this tissue due to its role in inhibiting Na^+^ transport. Therefore, a hypoxia-elicited increase in H_2_S restrains Na^+^ reabsorption-dependent oxygen expenditure, restoring oxygen balance [172].

### 8.2. Carbon Monoxide (CO)

Carbon monoxide is produced by heme oxygenases (HOs) through degradation of heme, which is associated with cofactors NADPH and cytochrome P450 reductase [173]. Endogenous CO is recognized as a physiological inhibitor of carotid body activity. Evidence indicates that exogenous utilization of CO at low concentrations inhibits the carotid body activity [174]. Studies implicate that increased carotid body chemo-reflex in the progression of autonomic morbidities is linked with cardiorespiratory diseases, like essential hypertension [175].

### 8.3. Nitric Oxide (NO)

Nitric oxide (NO) generation is considered as the functional status of endothelial cells. In addition to eNOS, as stated above, NO can be produced from cells in pathological conditions. Inflammation has been proven to be a factor in the pathophysiology of hypertension, which stimulates NO generation from immune cells [176]. It has been shown that human and animal hypertension present unchanged or reduced plasma NO levels, suggesting that inflammatory reactions might be responsible for the unchanged level of NO in the systemic circulation. Reduced levels of endothelium-derived NO generation might be regulated and balanced by NO that is secreted from immune cells [177,178].

### 8.4. Interaction of Biological Gases

Blood pressure regulation strongly relies on renal NO availability. Hypertension and chronic kidney disease (CKD) are linked with reduced NO levels in the kidney [179]. H_2_S and CO mutually play roles in controlling vascular function. It has been reported that the application of H_2_S contributes to cardio-protection by transverse aortic constriction through upregulation of eNOS and NO bioavailability [180]. H_2_S depletion consistently affects the other enzymes producing gas. At both time points, NO metabolites are decreased, and CO generation is greatly elevated. Moreover, the depletion of H_2_S decreases NO metabolites, whereas H_2_S is not decreased when NO is depleted. In general, endogenous H_2_S and CO are found to inhibit each other’s generation [181]. However, studies showed that inhibiting CSE accelerated CO production, whereas restraining HO-1, which regulated the availability of cellular heme, promoted H_2_S generation. The resulting high levels of CO have been found to inhibit NOS activity and NO generation [182]. Additionally, inhibition of eNOS is found to attenuate vasorelaxation stimulated by H_2_S [183].

H_2_S and NO metabolites during Sn(IV) protoporphyrin IX dichloride (SnPP) exposure were increased in the kidney, which is probably because of the reduction in CO binding to the heme cofactor of CBS and NO synthases, respectively [184]. Interestingly, inhibition of H_2_S- or CO-producing enzymes caused no effect on blood pressure and renal function [184]. The study also revealed that CO became redundant when sufficient NO and H_2_S were produced. Nevertheless, when the production of NO and H_2_S was inhibited, CO was enhanced as a compensatory mechanism. H_2_S was suggested to be a stronger regulator of CO than NO. CO can modulate NO generation and presents a compensatory relationship with H_2_S even though no direct interactions between NO and H_2_S are observed in the kidney. Hence, CO is the connecting factor between NO and H_2_S in the kidney [184].

## 9. Other Mechanisms

### 9.1. Asymmetric Dimethylarginine

Asymmetric dimethylarginine (ADMA) is an endogenous inhibitor of NO synthase (NOS), which contributes to oxidative stress and the development of hypertension. Low nephron numbers and an impaired ADMA-NO pathway are involved in programmed hypertension in the adult offspring of malnourished or diabetic mothers [185]. Decreased nephron numbers damage renal tubular sodium reabsorption, while the changed renin–angiotensin system (RAS) components interfere with sodium retention, which ultimately raises blood pressure and induces kidney damage.

### 9.2. Glucocorticoid Level

Excess glucocorticoid may be caused by increased expression of messenger (m) RNA and protein levels of the glucocorticoid receptor, which is accompanied by decreased gene expression of corticosteroid 11-β-dehydrogenase isozyme 2. Basically, corticosteroid 11-β-dehydrogenase isozyme 2 is the main enzyme converting cortisol into inactive cortisone, which therefore leaves the mineralocorticoid receptor unprotected and allows it to be alternatively activated. This alteration leads to increased blood pressure [186]. However, in a different position of VSMCs, complete methylation was observed in the eNOS promoter. Moreover, with regard to the alterations in methylation of the eNOS promoter in endothelial cells, the eNOS core promoter is concentrated in acetylated histones H3/K9 and H4/K12, and methylated H3/K4 [187]. After the application of trichostatin A, an HDAC inhibitor, eNOS expression was decreased in non-endothelial cells. Additionally, eNOS expression was suppressed by small RNA through changing histone acetylation and DNA methylation in endothelial cells [188].

### 9.3. Taurine

Taurine (2-aminoethanesulfonic acid) is a free amino acid, non-protein, presenting in many tissues, especially the brain, myocardium, liver, muscle, and kidney [189]. Taurine has shown the ability to reduce blood pressure. Dietary taurine is found to reduce hypertension in spontaneously hypertensive rats (SHRs) [190]. Furthermore, epidemiological studies suggest that the low incidence of cardiovascular disease is inversely related to dietary taurine intake [191]. In the study with respect to the renin–angiotensin system, taurine was able to inhibit cardiac hypertrophy in adult rats, which was induced by angiotensin II [192].

## 10. Conclusions and Perspectives

Increasing evidence indicates that epigenetic regulation has a huge potential for treating hypertension and renal diseases. Locus-specific epigenetic editing is likely to be the preferred therapeutic approach in the future. Nonetheless, the precise mechanisms underneath epigenetic phenomena have not been fully understood, especially at the molecular level, which, on the other side, leaves a great space for further research. It has been demonstrated that there is a pathophysiological connection between hypertension and renal injuries via the renal vascular system. This system can, however, be controlled or affected by epigenetic modifications, suggesting opportunities to develop novel therapeutic strategies. Under the physiological scope, the understanding of hypertension-related kidney damage, such as the dysfunction of the endothelium, nephron, and glomerulus, still stands at a preliminary level. Further studies are required to gain basic insights explaining the interactions among epigenetic marks and mediators. By discovering the missing jigsaw puzzle piece, a bridge between hypertension and renal dysfunction will hopefully be built, and the approaches for reversing the remodeling of the relevant physiological units will be developed.

## Abbreviations

CCT, cortical collecting tubule; c-Myc, Myelocytomatosis; CTCF, CCCTC-binding factor; GATAs, transcription factors of zinc finger DNA-binding proteins; GNAT, Gcn5-related N-acetyltransferase; HBO1 is an acetyltransferase; HDAC, histone deacetylase; HIF-1α, Hypoxia-inducible factor-1α; MBD, methyl-CpG-binding domain protein; MeCP2, methyl-CpG-binding protein 2; the MYST, protein family are catalytic subunits of histone acetyltransferase (HAT) complexes; NOTCH is a family of type 1 transmembrane proteins that form a core component of the Notch signaling pathway; NRF1, Nuclear respiratory factor 1; PCT, proximal collecting tubule; SM22α—is a calponin-related protein that is expressed specifically in adult smooth muscle.
